# The changing landscape for the management of patients with neovascular AMD: brolucizumab in clinical practice

**DOI:** 10.1038/s41433-022-02008-3

**Published:** 2022-03-21

**Authors:** Ian Pearce, Winfried Amoaku, Clare Bailey, Louise Downey, Richard Gale, Faruque Ghanchi, Robin Hamilton, Sajjad Mahmood, Geeta Menon, Jenny Nosek, James Talks, Yit Yang

**Affiliations:** 1 St Paul’s Eye Unit, Royal Liverpool University Hospital, Liverpool, UK; 2 Academic Ophthalmology, Division of Clinical Neuroscience, University of Nottingham, Nottingham, UK; 3 University Hospitals Bristol and Weston NHS Foundation Trust, Bristol, UK; 4 Hull University Teaching Hospital, Hull, UK; 5 University of York and York Teaching Hospital, York, UK; 6 Bradford Teaching Hospitals NHS Foundation Trust, Bradford, UK; 7 NIHR Biomedical Research Centre at Moorfields Eye Hospital NHS Foundation Trust, London, UK; 8 Faculty of Biology, Medicine and Health, University of Manchester, Manchester, UK; 9 Frimley Health NHS Foundation Trust, Frimley, UK; 10 Royal Shrewsbury Hospital, Shropshire, UK; 11 Newcastle upon Tyne Hospitals NHS Foundation Trust, Newcastle upon Tyne, UK; 12 Wolverhampton Eye Infirmary, The Royal Wolverhampton NHS Trust, Wolverhampton, UK

**Keywords:** Macular degeneration, Education

## Abstract

Untreated neovascular age-related macular degeneration (nAMD) can lead to severe and permanent visual impairment. The chronic nature of the disease can have a significant impact on patients’ quality of life and an economic and time burden on medical retina (MR) services, with the care need outweighing the growth of resources that clinical services can access. The introduction of a new treatment into clinical services can be challenging, especially for services that are already under capacity constraints. Guidance for practical implementation is therefore helpful. Roundtable meetings, facilitated by Novartis UK, between a working group of MR experts with experience of leading and managing NHS retinal services in the intravitreal era were conducted between 2020 and 2021. These meetings explored various aspects and challenges of introducing a new anti-vascular endothelial growth factor (VEGF) therapy to the UK medical retina services. Provision of clear expert recommendations and practical guidance nationally, that can be adapted locally as required to support clinicians and healthcare professionals (HCPs), is valuable in supporting the introduction of a new anti-VEGF therapy within the NHS environment. The experts provide ophthalmologic HCPs with a collation of insights and recommendations to support the introduction and delivery of brolucizumab in their local service in the face of current and projected growth in demand for retina care.

## Abstract (zh)

未经治疗的新生血管年龄相关性黄斑变性(nAMD)可导致严重永久性的视力损害。该疾病的慢性进展性的临床特征会对患者的生活质量产生重大影响, 并对视网膜疾病的服务造成经济方面和时间方面的负担, 其护理需求超过了临床服务目前可获得的资源增速。在临床服务中引入一种新的治疗方法可能具有挑战性, 特别是对于已经受到限制的服务而言。因此, 对临床实践进行指导是非常有益的。在诺华英国公司的协助下, MR专家工作组在2020年至2021年期间召开了圆桌会议, 这些专家拥有在玻璃体腔注药时代领导和管理NHS视网膜服务的丰富经验。这些会议探讨了将一种新的抗血管内皮生长因子(VEGF)引入英国医疗视网膜服务的各个方面的问题和挑战。为了支持临床医生和医疗保健专业人员(healthcare professionals, HCPs), 对在全国范围内提供的明确的专家建议和实用指南, 可以根据需要在当地进行调整, 这对于支持在NHS环境中引入新的抗VEGF疗法非常有价值。面对当前和预计的视网膜护理需求增长, 专家们为眼科医务人员提供了一系列见解和建议, 以支持在他们的本地服务中引入和使用布洛赛珠单抗。

## Introduction

Age-related macular degeneration (AMD) is the leading cause of blindness in developed countries [1–4]. Neovascular AMD (nAMD) is the third leading cause of blindness worldwide, after cataracts and glaucoma [5]. If left untreated, the progressive loss of central visual acuity (VA) that characterises nAMD leads to severe and permanent visual impairment and certified vision loss [4, 6–8].

The chronic, progressive nature of the disease can have a significant impact on patients’ quality of life, imposing substantial time burden, limiting their ability to perform day-to-day tasks and have a significant emotional impact [4, 9]. Insufficient medical retina service capacity, delays in treatment initiation and poor patient adherence can all contribute to suboptimal outcomes for patients [4, 9, 10]. The progressive nature of the disease means that as time passes the economic and time burdens for caregivers and services increase [9], with the care need outweighing the growth of the resources allocated to clinical services [10]. The need for continued treatment and monitoring results in a significant financial impact on the health service [11].

The current mainstay of treatment for nAMD remain anti-vascular endothelial growth factor (anti-VEGF) therapies [12–15]. The introduction of intravitreal injections of anti-VEGF resulted in a paradigm shift for nAMD treatment [16, 17], following the results from pivotal trials of anti-VEGF therapies that have demonstrated their efficacy in improving VA in patients with nAMD [12, 13, 18–23]. These clinical trials and their associated extension studies show that initial VA gains can be maintained for up to 7 years with continuous proactive anti-VEGF treatment [13, 14, 24, 25].

However, in the real-world the early VA gains typically decline both in short-term and in the longer-term studies [26–29], often as a result of patients being under-treated [30]. The causes for such a decline could include the burdens for both the patients (adherence, frequent injections and monitoring visits acting together to impact compliance) and clinicians (logistical and time burdens on the clinic) [30]. In addition, services currently have the added burden of the COVID-19 pandemic, which has had a major impact on healthcare provision and reorganisation of outpatient clinics to mitigate COVID-19 risk, impacting the ability to deliver effective nAMD clinics [31].

Brolucizumab is a monoclonal antibody, which binds VEGF-A and as a result reduces neovascularisation and vascular permeability [32, 33]. Brolucizumab (6 mg) has been assessed in two phase 3, randomised clinical trials (RCT), HAWK and HARRIER, which used an anti-VEGF (aflibercept) as an active control in 1088 treatment-naive nAMD patients [19, 20].

In both HAWK and HARRIER, at Week 48, brolucizumab (6 mg) demonstrated noninferiority to aflibercept in best-corrected VA (BCVA) change from baseline (6.6 and 6.9 vs. 6.8 and 7.6, respectively) [20]. These robust VA gains were maintained to Week 96 (5.9 and 6.1 letters respectively) [19]. When assessed at 16 weeks fewer patients treated with brolucizumab (6 mg; 24% and 22.7%) had disease activity compared with aflibercept (34.5% and 32.2%) [20]. Greater central subfield thickness (CST) reductions were observed with brolucizumab (6 mg) compared with aflibercept in both HAWK and HARRIER at both Week 48 and 96 [19, 20]. In addition, the proportion of eyes with intraretinal fluid (IRF) and/or sub-retinal fluid (SRF) at Week 48 was significantly lower in eyes treated with brolucizumab (6 mg) compared with aflibercept and this difference was maintained at week 96 [19, 20].

As a consequence of the positive registration studies and risk:benefit ratio, brolucizumab received market authorisation from European Medicines Agency and is recommended by the National Institute for Health and Care Excellence (NICE) and accepted by Scottish Medical Council for the treatment of patients with nAMD [32, 33].

Introducing a new treatment into the clinic for any disease can be a challenging task and is particularly challenging in services that are already under significant capacity constraints (volume of patients, injections, monitoring, resource). Consequently, the provision of guidance for practical implementation of brolucizumab to UK services is necessary. A working group of MR experts with experience of leading and managing NHS retinal services in the intravitreal era covering a reasonable geographic spread across England recently discussed (meetings between February 2020 and August 2021) the key aspects of delivering anti-VEGF therapy and, in particular, brolucizumab. The aim was to provide expert opinion and guidance on the practical implementation of brolucizumab into UK medical retina services. This article presents a collation of insights, guidance and resources to help support all ophthalmologic healthcare professionals (HCPs) with the necessary tools to deliver brolucizumab in their local service in the face of current and projected growth in demand for retina care.

## Patient populations

### Recommended patient population

Brolucizumab is recommended for patients with a confirmed diagnosis of nAMD [32, 33]. In patients with nAMD who are naive to treatment with anti-VEGF agent, it is advised that all the NICE-recommended options for nAMD are discussed with them [15, 34, 35]. Provided there are no contraindications, intravitreal anti-VEGF treatment should be initiated promptly (Tables 1 and 2) [32, 36, 37].

**Table 1:** Contraindications for intravitreal anti-VEGF therapies [32, 36, 37].

Contraindications for ALL anti-VEGF therapies	• Hypersensitivity to active substance or any excipients. • Patients with active or severe intraocular inflammation (IOI) [previous history of anterior uveitis is not considered an exclusion criterion]. • Active or suspected ocular or periocular infections.

**Table 2:** Specific factors to consider for intravitreal brolucizumab treatment.

Cautionary criteria for brolucizumab based on clinical experts’ opinion	• History of any intraocular inflammation (IOI). • Patients with prior history of retinal vasculitis. • Eyes with scleritis and episcleritis may be excluded at the discretion of the treating physician. • Previous culture-negative endophthalmitis. • The evidence on efficacy and adverse events of brolucizumab is continually evolving; there is a need to review the data and seek advice as necessary from lead or senior consultants.

Although not studied within the phase 3 registration studies, in eyes already receiving treatment with an intravitreal anti-VEGF agent, brolucizumab may be an appropriate treatment option in patients where disease control has not been achieved and/or where treatment interval extension fails or remains lower than desired [38].

In the experts’ opinions, patients requiring bilateral treatment may receive either sequential brolucizumab injections or bilateral simultaneous injections (ensure different batches of anti-VEGF are used if same day injection), based on the physician’s expertise, preference and following discussion with the patient. However, the safety and efficacy of brolucizumab administered in both eyes concurrently has not been studied [32]. In clinical practice, patient selection is important for optimising outcomes and minimising adverse events (AEs). It is important to consider the risk vs. benefit for patients in only eyes and bilateral disease.

Whilst any new patient with progressive nAMD is potentially eligible for brolucizumab a practical stratified introduction of brolucizumab in clinical practice may be considered. For example, there may be a particular benefit for patients keen to avoid frequent injections or frequent visits to the clinic or patients who may have difficulty attending regular fundus examinations. This should be balanced against the very low risk of return to the eye department and potential treatment needs due to IOI. Further clinical evidence exploring different treatment regimens (e.g., treat to control in the TALON study [39]) and the generation of real-world experience is ongoing and will further support the use of brolucizumab in clinical practice.

## Risk vs. benefit considerations

When considering the use of brolucizumab part of the discussion regarding informed consent will need to include the benefit vs risk. The potential benefit of brolucizumab for the treatment of patients with nAMD has been demonstrated in the HAWK and HARRIER, randomised, phase 3 clinical trials [19, 20].

**Fig. 1:**
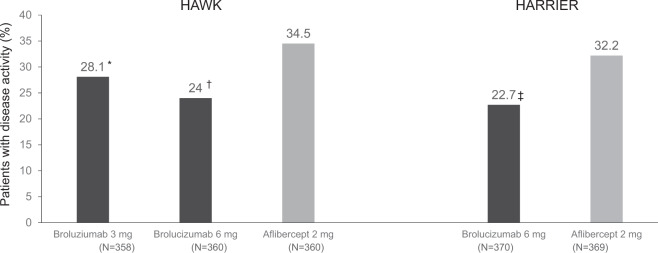
Disease activity at week 16 in HAWK and HARRIER. Full analysis set; analysis conducted based on the efficacy/safety approach. *The 95% confidence interval (CI) for treatment difference, –13.2 to 0.3; *P* = 0.033. ^†^95% CI for treatment difference, –17.1 to –3.5; *P* = 0.001. ^‡^95% CI for treatment difference, –15.8 to –3.1; *P* = 0.002. One-sided *P* values versus aflibercept.

In these trials, brolucizumab demonstrated noninferiority with respect to BCVA change from baseline at Week 48 (least squares [LS] mean, +6.6 [6 mg] letters with brolucizumab vs. +6.8 letters with aflibercept [HAWK]; + 6.9 [brolucizumab 6 mg] vs. +7.6 [aflibercept] letters [HARRIER]; *P* < 0.001 for each comparison) [20]. The mean change (LS mean ± standard error) in BCVA from baseline to 96 weeks was maintained in HAWK (5.90 ± 0.78 letters for brolucizumab 6 mg, and 5.3 ± 0.78 letters for aflibercept) and in HARRIER (6.1 ± 0.73 letters for brolucizumab 6 mg and 6.6 ± 0.73 letters for aflibercept) [19]. In terms of fluid in the macula, eyes treated with brolucizumab (6 mg) had greater CST reductions throughout the duration of the study and significantly lower IRF and/or SRF compared to aflibercept [19, 20]. In addition, at Week 16, after identical treatment exposure, fewer brolucizumab–treated eyes (6 mg) had disease activity compared to aflibercept in HAWK (24.0% vs. 34.5%; *P* = 0.001) and HARRIER (22.7% vs. 32.2%; *P* = 0.002, Fig. 1). This better control of fluid would be expected to lead to a reduced frequency of injections using a personalised regimen such as Treat and Extend and may suggest the possibility of better fluid control in patients already on optimal treatment with an existing anti-VEGF but with persistent fluid.

Also of note, in both trials, approximately half the enroled eyes receiving brolucizumab (6 mg) were maintained on a 12-weekly (Q12W) dosing interval through to Week 48 immediately following the initial three 4-weekly loading doses [20]. Importantly, eyes treated with brolucizumab that did not require a treatment between the final loading injection and the first Q12W injection were likely to be maintained on a Q12W injection interval for the study, demonstrating a decreased disease activity and durability with brolucizumab [19, 20].

An important reason for establishing a patient profile is to avoid treating those who may be susceptible to the AEs identified during clinical trials of brolucizumab [32]. Even with careful profiling, however, any pharmacological treatment carries a risk of unwanted or unexpected AEs [40]. This is why healthcare bodies such as the Medicines and Healthcare products Regulatory Agency (MHRA) compile post-marketing reports of side effects and adverse drug reactions [40].

Injection site reactions (ISRs) are a local phenomenon and are one of the most commonly occurring AEs following administration of a drug or vaccine via injection [41]. ISRs can include swelling, erythema, pruritus and pain at the injection site [41]. In most cases, ISRs are mild to moderate and resolve after a few days. In addition to the risk of reaction from the injection itself, reactions may also occur from the drug injected. These AEs are extensively studied in clinical trials and are included in the Summary of Product Characteristics (SmPC) and patient information leaflet (PIL) to help clinicians and patients make informed decisions about their care [32, 42].

### Adverse events with anti-VEGF treatments in nAMD

The availability of multiple anti-VEGF therapies, each with different molecular configurations, creates unique challenges to mapping treatment responses [43]. This is compounded by individual patient and disease characteristics—such as age, lesion characteristics and lesion duration—in addition to variations in injection frequency observed between clinical centres delivering the same agent [43, 44]. Despite variation in treatment responses to anti-VEGF therapies, potential AEs are similar across the different agents [45].

Each intravitreal injection with an anti-VEGF therapy in patients with nAMD is associated with a risk of post-injection and drug-class-associated AEs (Table 3) [45–47].

**Table 3:** Adverse events associated with intraocular anti-VEGF injections.

Endophthalmitis	• A complication of intravitreal injections resulting from an infection [45, 46]. • An inflammation of the internal eye tissues, which can cause redness, sensitivity to light and pain [46]. If treatment is not started promptly, patients may suffer reduction or loss of vision [46].
Intraocular inflammation (IOI)	• One of the main adverse events associated with anti-VEGF injections, incidence varies between the various therapies [45]. IOI can be limited to anterior uveitis but when it involves posterior segments and especially if it is severe, in clinical practice, it may be difficult to distinguish between endophthalmitis and IOI [45]. Occlusive vasculitis is a sight-threatening form of IOI and has been described in association with brolucizumab use.
Rhegmatogenous retinal detachment	• There is low incidence of rhegmatogenous retinal detachment following administration of anti-VEGF therapies (0–0.67%) and risk factors include incorrect injection technique [45, 47].
Intraocular pressure elevation	• The rise of intraocular pressure has been associated with the intravitreal injection procedure and is likely to last, at most, a few hours [45]. However, studies have linked anti-VEGF therapies to an increased risk for intraocular pressure elevation [45].
Ocular haemorrhage	• A number of reports have been published regarding ocular haemorrhage following administration of anti-VEGF therapies [45]. This includes subconjunctival haemorrhage, reported in ~10% of injections, and instances of massive choroidal detachment, and massive sub-retinal haemorrhage associated with specific therapies [45].
Systemic complications	• Systemic administration of anti-VEGF has been linked to severe adverse reactions, including thromboembolic events, myocardial infarction, stroke and hypertension [45]. • All intravitreal anti-VEGF injections have reported detectable levels of systemic circulation, and so caution should be taken to monitor potential systemic adverse events [45].

### Safety profile for brolucizumab

The most common AEs reported in the pivotal Phase 3 trials, HAWK and HARRIER, included cataract, conjunctival haemorrhage, reductions in visual acuity and vitreous floaters (Table 4) [20].

**Table 4:** Ocular adverse events reported in HAWK and HARRIER [20].

Preferred term	HAWK, *n* (%)			HARRIER, *n* (%)	
	Brolucizumab 3 mg (*n* = 358)	Brolucizumab 6 mg (*n* = 360)	Aflibercept 2 mg (*n* = 360)	Brolucizumab 6 mg (*n* = 370)	Aflibercept 2 mg (*n* = 369)
No. of patients with at least 1 event	218 (60.9)	220 (61.1)	201 (55.8)	174 (47.0)	176 (47.7)
Conjunctival haemorrhage	39 (10.9)	29 (8.1)	32 (8.9)	17 (4.6)	19 (5.1)
Visual acuity reduced	34 (9.5)	22 (6.1)	29 (8.1)	32 (8.6)	26 (7.0)
Vitreous floaters	26 (7.3)	22 (6.1)	16 (4.4)	15 (4.1)	5 (1.4)
Retinal haemorrhage	14 (3.9)	21 (5.8)	20 (5.6)	12 (3.2)	4 (1.1)
Cataract	18 (5.0)	20 (5.6)	13 (3.6)	11 (3.0)	43 (11.7)
Vitreous detachment	24 (6.7)	19 (5.3)	19 (5.3)	10 (2.7)	8 (2.2)
Dry eye	20 (5.6)	19 (5.3)	26 (7.2)	10 (2.7)	11 (3.0)
Eye pain	28 (7.8)	18 (5.0)	21 (5.8)	13 (3.5)	19 (5.1)
Posterior capsule opacification	16 (4.5)	14 (3.9)	11 (3.1)	7 (1.9)	5 (1.4)
Intraocular pressure increased	16 (4.5)	13 (3.6)	15 (4.2)	14 (3.8)	15 (4.1)
Blepharitis	8 (2.2)	13 (3.6)	12 (3.3)	13 (3.5)	5 (1.4)
Retinal pigment epithelial tear	5 (1.4)	12 (3.3)	4 (1.1.)	8 (2.2)	5 (1.4)
Vision blurred	16 (4.5)	11 (3.1)	10 (2.8)	3 (0.8)	3 (0.8)
Visual impairment	15 (4.2)	10 (2.8)	14 (3.9)	1 (0.3)	3 (0.8)
Eye irritation	10 (2.8)	10 (2.8)	11 (3.1)	4 (1.1)	1 (0.3)
Punctate keratitis	11 (3.1)	9 (2.5)	10 (2.8)	1 (0.3)	7 (1.9)
Conjunctivitis	3 (0.8)	9 (2.5)	3 (0.8)	15 (4.1)	8 (2.2)
Iritis	3 (0.8)	9 (2.5)	1 (0.3)	0 (0.0)	1 (0.3)
Uveitis	6 (1.7)	8 (2.2)	1 (0.3)	3 (0.8)	0 (0.0)
Visual field defect	9 (2.5)	7 (1.9)	5 (1.4)	1 (0.3)	0 (0.0)
Corneal abrasion	6 (1.7)	7 (1.9)	10 (2.8)	1 (0.3)	1 (0.3)
Macular fibrosis	10 (2.8)	5 (1.4)	4 (1.1)	3 (0.8)	1 (0.3)
Dry age-related macular degeneration	7 (2.0)	5 (1.4)	3 (0.8)	7 (1.9)	4 (1.1)
Foreign body sensation in eyes	8 (2.2)	4 (1.1)	9 (2.5)	1 (0.3)	4 (1.1)
Lacrimation increased	7 (2.0)	4 (1.1)	5 (1.4)	4 (1.1)	3 (0.8)
Lenticular opacities	7 (2.0)	1 (0.3)	4 (1.1)	13 (3.5)	12 (3.3)

Of the AEs reported, there were three key observations of interest, which included: [48]

- IOI
- Retinal vasculitis
- Concomitant retinal vasculitis and retinal occlusion

Following the occurrence of 70 IOI-related AEs in HAWK and HARRIER, 87.1% (*n* = 61) were treated [49]. The majority of patients received topical corticosteroids, while a minority received systemic and intraocular corticosteroids [49]. Inflammation resolved completely in 79.6% of eyes (*n* = 39), resolved with sequelae in 10.2% of eyes (*n* = 5) and did not resolve in 10.2% of eyes (*n* = 5) by the end of the study [49].

The 2-year risk of definite (28/50 patients) and probable (22/50 patients) IOI, retinal vasculitis and/or retinal occlusion following treatment with brolucizumab are outlined in the table below, following a review of observations of interest by an independent Safety Review Committee (SRC), supported by Novartis to analyse these investigator-reported cases in the phase 3 HAWK and HARRIER trials (Table 5) [48].

**Table 5:** Two-year risk of intraocular inflammation, retinal vasculitis and/or retinal occlusion following treatment with brolucizumab [48].

Observations of interest (IOI, retinal vasculitis [RV] and/or retinal vascular occlusion [RVO])	Overall risk of developing IOI, vasculitis or RVO	Overall risk of developing at least moderate vision loss (≥15 ETDRS letter loss)^a^	Subpopulation risk of developing at least moderate vision loss (≥15 ETDRS letter loss)^a^	Overall risk of developing severe vision loss (≥30 ETDRS letter loss)^b^	Subpopulation risk of developing severe vision loss (≥30 ETDRS letter loss)^b^
50 patients developed IOI with or without vasculitis and with or without RVO	4.6% (50/1088)	0.7% (8/1088)	16.0% (8/50)	0.5% (5/1088)	10% (5/50)
36 of the 50 patients with IOI had RV	3.3% (36/1088)	0.7% (8/1088)	22.2% (8/36)	0.5% (5/1088)	13.9% (5/36)
23 of the 36 patients with IOI and RV had RVO	2.1% (23/1088)	0.6% (7/1088)	30.4% (7/23)	0.5% (5/1088)	21.7% (5/23)

^a^Eight patients with vasculitis developed moderate vision loss; 7 of the 8 also had retinal vascular occlusion.

^b^Five patients with vasculitis and retinal vascular occlusion developed severe vision loss.

Overall, fifty patients (4.6%) developed IOI, of which 26 (3.3%) developed retinal vasculitis and 23 (2.1%) developed concomitant retinal vasculitis and retinal occlusion [30, 48]. Over two years, the absolute risk of developing IOI of any form was 4.6%, and the risk of visual loss due to IOI (≥15 letters) was 0.7% [30, 48]. The overall rate of vision loss was similar when comparing the brolucizumab and aflibercept arms of the study population [48].

The majority of inflammatory events (74%, 37/50) occurred in the first 6 months following the first dose of brolucizumab [48]. Additionally, nearly half of these events occurred within the first 3 months (48%, 24/50) [30]. Post-hoc analysis of HAWK and HARRIER reported that, of the 8 cases of vision loss ≥15 ETDRS letters in eyes with IOI, 5 patients experienced their first IOI-related event within 3 months of the first brolucizumab injection [30]. By 6 months, this increased to 7 out of the 8 patients [30].

Of the 73.5% (36/49) eyes that continued receiving brolucizumab following the first IOI-related AE, 24 completed the study and 12 discontinued [49]. Mean BCVA change in these eyes was 7.8 and –7.7 ETDRS letters, respectively, from baseline to the end of the study [49]. 26.5% (13/49) eyes were not treated with brolucizumab following the first IOI-related AE and the mean BCVA change was—10.4 ETDRS letters from baseline to the end of the study [49].

The MERLIN phase 3 study was designed to compare safety and efficacy of brolucizumab 6 mg dosed every 4 weeks to aflibercept 2 mg dosed every 4 weeks in those nAMD patients with retinal fluid despite frequent anti-VEGF injections [50]. In this study, brolucizumab administered every 4 weeks demonstrated a higher incidence of IOI-related AEs with more frequent dosing compared with aflibercept [51]. However, these are the first interpretable results and further analysis of the data is required. Furthermore, MERLIN also adopted a 4-week injection interval following the first three initiation doses. However, the current posology and data from the clinical trials (HAWK/HARRIER) are based on injections every 8 or 12 weeks depending on disease activity after the 3 initiation doses [20, 32]. Given the data from the MERLIN study, it is recommended that patients should have a minimum 8-week interval between injections once the loading phase has been completed.

A recent cohort study of patients from the Intelligent Research in Sight (IRIS) Registry (US eye disease database) and Komodo Healthcare Map (US claims database) has examined real-world evidence of IOI, including retinal vasculitis (RV) and/or retinal vascular occlusion (RO) in patients with nAMD who received brolucizumab treatment between October 8, 2019, and June 5, 2020 [52]. The analysis include over 21,000 treated eyes, with the majority being switched from another anti-VEGF therapy [52]. In this cohort analysis, the overall incidence of IOI and/or RO was 2.4% in each registry. Also of note is that patients with a history of IOI and/or RO in the preceding 12 months had an increased observed risk rate (8.7% [95% CI, 6.0–11.4%] and 10.6% [95% CI, 7.5–13.7%]) for an IOI and/or RO event in the 6 months following the first brolucizumab treatment compared with patients without prior IOI and/or RO (2.0% in both data sets) [52]. However, due to the nature of the design of the studies, there are limitations to using the analysis of the risk factors as predictors of the rates of IOI and/or RO events [32, 52].

Data regarding IOI following brolucizumab administration is constantly evolving and will likely change in the future as experience and evidence increases.

### Mitigating and managing adverse events

Several approaches can be used to mitigate possible AEs following administration of brolucizumab [53]. The most conservative approach to monitoring is to have patients return between injections for safety assessments; however, this will reduce the benefit vs. risk ratio due to the increased burden on the service. Another option is to allow patients to self-monitor for symptoms between injection visits. The most pragmatic approach is for patients to be monitored and assessed for IOI at each scheduled visit in conjunction with educating the patient to self-monitor. Clinical management recommendations include; slit-lamp biomicroscopy (for anterior chamber inflammatory activity), dilated fundus examination, colour fundus photography and optical coherence tomography (OCT) scan (for the detection of vitreous cells and evaluation of the retina for signs of inflammation). Also consider (if available) ultra-widefield (UWF) imaging and fluorescein angiography as helpful adjunctive investigational tools [53].

Assessments for side effects, visual acuity and anatomy on OCT scans and appropriate detailed questioning of patients are recommended at each treatment visit to help optimally guide future treatment. Intermediary assessment visits are not required in the intervals between treatment visits unless the patient presents as an emergency with new visual symptoms. Reports indicate that 75% of patients who had inflammatory side effects to brolucizumab did so in the first six months [20, 49]. This offers some reassurance to the patient and clinician that risk is reduced after six months on treatment but in view of the small ongoing risk, checks for inflammation and RO are recommended at each visit.

Educating patients on the importance of reporting symptoms as soon as they arise can be vital in ensuring an early diagnosis [53]. This can include asking the patients specific questions about any changes they observed after injection and ensuring they know what to look out for (change in vision) between injection visits and providing patient materials to support them.

Careful monitoring, prompt diagnosis and timely intervention are key to managing potential AEs associated with brolucizumab [53]. Upon diagnosis of IOI, RV or a RO, treatment with brolucizumab should be discontinued [32, 53]. Intensive treatment of IOI with corticosteroids, followed by regular monitoring, may help in preventing IOI progression [53].

Recent studies have identified several risk factors for IOI, including neutralising antibodies (Nab), female gender and prior history of IOI [54, 55]. In the phase 3 trial HAWK and HARRIER, 86% of patients with IOI and RV had demonstrated Nab prior to, or at the time of, the AE [54]. Nab and their involvement in inflammation following treatment with brolucizumab is being explored to enable us to provide better advice in the future. However, the information available to date does not provide any clinically relevant predictive tests for IOI. Further studies will be needed to develop better understanding of these risk factors and whether they can be used when deciding which patients should receive treatment with brolucizumab.

## Informed patient consent

Appropriate informed consent should be obtained before the start of any anti-VEGF treatment, including brolucizumab. In real-world clinical practice, one of the most important parts when evaluating a patient’s suitability is the risk vs. benefit profile of the treatment.

A recent publication, ‘NHS Resolution best practice guide for clinicians and managers’ (Getting It Right First Time May 2021) [56] supports the principles of the ‘three-legged stool’ approach to patient consent, proposed by the British Association of Spinal Surgeons [57]. This model consists of three distinct aspects to consent that support the consenting process, and should be considered when discussing brolucizumab to ensure patients are well informed and are supported to make the best decisions about their treatment pathway:

### Patient-centred dialogue

Patients obtain information relating to their condition and help form good clinician partnerships through face-to-face conversations. These conversations are particularly important in nAMD where patients are required to attend regular clinics for treatment and monitoring. These conversations are also useful for broader conversations with the patient to ensure they have been informed of all the risks vs. benefits as individual patients will accept different levels of risk. This discussion should include the reason one anti-VEGF is considered over another.

This patient-centred dialogue is essential in the different clinics, for example in a 1-stop service where patients have been directly referred to the nAMD clinic this is the first opportunity to discuss the treatment options and outline why brolucizumab is being considered over other anti-VEGF options. While in a 2-stop service patients would likely receive written information and may have had time to reflect on the information they’ve received.

In addition, verbal or written information should include information that the proportion of eyes that lost 15 letters or more by the end of two years was comparable between the brolucizumab and aflibercept treated groups [20].

**Table:** 

*Benefit vs. risk discussion between patient and clinician regarding brolucizumab*
During the process of obtaining patient consent to undergo treatment with brolucizumab, it is important to discuss the benefits (such as potentially fewer injections), balanced with the potential AEs (such as the potential risk for inflammation) and put this information into context e.g., explaining the overall risk of vision loss is not per injection but based on two years of treatment as per the clinical trials. This should also be included in patient materials.It is important to counsel patients that, with careful monitoring and early detection, most symptoms of IOI associated with brolucizumab can be appropriately managed and rarely results in loss of vision [58]. Provision of an emergency contact number to patients is essential. Patients should be advised to report, as a matter of urgency, if they experience the following symptoms: sudden decrease or change in their vision, including an increased number of small particles or flashes of light; increasing pain or worsening redness in the eye; increased sensitivity to light.

### Patient information booklet

Patients are required to take in large amounts of information to make an informed decision during the informed consent process; supporting information to remind patients of what has been discussed is therefore vital to assist with retaining that information. Information on anti-VEGFs needs to be written in patient-friendly language and be consistent with what their doctor has discussed, including a clear explanation of the risks and benefits. Ideally, the patient should be given adequate time to digest the information and engage the support of their family or carer before deciding about their treatment. Providing information on anti-VEGFs for patients to take away from the consultation should help enable patients to take an active role in the decision-making process. Physicians can access relevant information via the emc website (medicines.org.uk).

### Procedure specific surgeon-guided consent form

When managing a medical retina service all healthcare organisations must have robust policies and procedures in place to ensure written patient consent is obtained appropriately and are also mandated to follow local policies. An example of a standardised consent form is provided in the appendix, which provides an example of a brolucizumab consent that covers previously discussed NHS directives [58].

## Treatment pathway

**Fig. 2:**
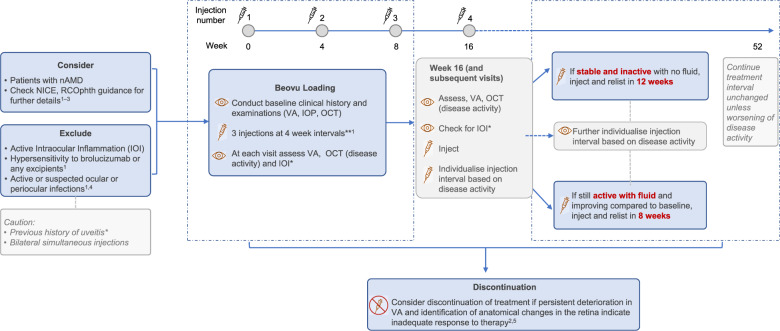
Brolucizumab treatment framework for patients with nAMD. *For further information see expert opinion for guidance. **Recommended dose is 6 mg brolucizumab (0.05 ml solution). BCVA best-corrected visual acuity; DA, disease activity, ETDRS early treatment diabetic retinopathy study, IOI intraocular inflammation, IOP intraocular pressure, nAMD neovascular age-related macular degeneration, OCT optical coherence tomography, RO retinal vascular occlusion, VA visual acuity. 1. Beovu SmPC. Available at: https://www.ema.europa.eu/en/documents/product-information/beovu-epar-product-information_en.pdf. Accessed February 2021; 2. NICE. TA672. Available at: https://www.nice.org.uk/guidance/TA672. Accessed February 2021; 3. RCOphth. AMD Services Commissioning Guidance Available at: https://www.rcophth.ac.uk/wp-content/uploads/2021/02/AMD-Commissioning-Guidance-Consultation.pdf. Accessed February 2021; 4. Zarbin M, et al. American Academy of Ophthalmology Annual Meeting, 13–15 November 2020; 5. German Society of Ophthalmology (Deutsche Ophthalmologische Gesellschaft, DOG); German Retina Society (Retinologische Gesellschaft e. V, RG); Professional Association of German Ophthalmologists (Berufsverband der Augenärzte Deutschlands e. V, BVA) *Ophthalmologe* 2021;118(Suppl 1):31–39.

Wherever possible brolucizumab should fit into the existing pathways in use by the service. The recommended treatment pathway for brolucizumab is based on the protocol used in the HAWK and HARRIER studies (Fig. 2) [19, 20]. These studies only included patients who were naive to intravitreal anti-VEGF treatment. The best evidence for the use of brolucizumab is therefore currently in treatment-naive eyes. Nevertheless, patients switching to brolucizumab from another anti-VEGF are likely to represent the main group of patients treated initially. It is therefore important to propose brolucizumab treatment pathways for both treatment-naive and switch patients.

### Treatment-naive patients

For treatment-naive patients, the first three injections—commonly referred to as ‘loading’ or ‘initiation’ doses – are administered at 4-week intervals (at weeks 0, 4 and 8) [20, 32]. After the third injection, the patient is scheduled for review 8 weeks later (week 16). A patient examination is recommended at each visit to check treatment effect as well as side effects. The patient should be asked about subjective changes in their vision, including loss of vision and new scotomata, pain, red eye, photophobia and symptoms of floaters. Asking for these specific symptoms at each treatment visit may promote patient vigilance for the symptoms of IOI and so lead to earlier self-referral between scheduled treatment visits should IOI occur. These symptoms should be included within a PIL given at the start of treatment.

Given the differences in services, not all clinics will have the ability to perform UWF imaging but if available this is an option for recording and monitoring peripheral retinal changes. Slit lamp biomicroscopy assessment for anterior chamber inflammatory activity is the gold standard and should be used, with or without UWF fundus imaging. Fundus examination of the eye after pupil dilation enables examination of the retina to check for signs of posterior segment inflammation and vascular occlusion. OCT scan can be used for the detection of vitreous cells and evaluation of the vitreomacular interface (VMI). Fluorescein angiography may be required if RV or occlusion is suspected.

A ‘disease activity’ assessment is advised at week 16 to determine the length of the interval between subsequent reviews. As a pragmatic approach and for consistency this assessment can be considered for each face-to-face visit. Visual acuity and OCT scan are used to determine disease activity. Visual acuity in isolation is not sufficient because of test-retest variability; nevertheless, stable or improved visual acuity is expected in a patient responding to treatment. Some reduction in vision may also occur in the presence of anatomical improvement and disease stabilisation; this should prompt investigation to rule out other causes than the patient’s AMD or refractive error. Anatomical features used to assess activity include fluid levels—intraretinal, sub-retinal, sub-retinal pigment epithelium. Changes in sub-retinal hyper-reflective material (SHRM) and macular haemorrhage are also used to assess activity. If there is no disease activity at week 16, the review interval may be extended to 12 weeks; this should remain at 12 weeks unless disease activity increases. In a patient with signs of improvement in disease activity (but not inactive) at week 16, treatment should be maintained at an 8-week interval. If the disease has worsened at week 16 compared with baseline, further investigations to re-evaluate the primary diagnosis and confirm the presence of nAMD should be considered.

Patients maintained on an 8-week treatment interval who have persistent nAMD activity can be assessed earlier (e.g., 4 weeks after a brolucizumab injection) to demonstrate that a good treatment effect is occurring, but then inadequate control occurs within an 8-week interval. These patients should be counselled that the minimum injection interval with brolucizumab after the loading phase is 8 weeks and reiterate that 4-weekly injections is associated with a higher incidence of IOI. The option then would be to consider switching to an alternative anti-VEGF agent that facilitates more frequent than 8-week dosing.

With this type of treat-and-extend approach, patients who achieve eventual complete disease control with dosing every 8 weeks can be extended to fixed 12-week intervals. Clinicians may consider potential extension beyond 12 weeks with monitoring, although evidence is currently lacking. The treatment interval may be titrated according to ongoing response to treatment and disease activity after each visit with a maximum interval of 12 weeks and minimum interval of 8 weeks at the treating clinician’s discretion.

If a patient shows increased disease activity when the treatment interval is extended, the treatment interval should be reduced back to the previous interval at which control was achieved. Repeated attempts to challenge at longer intervals may be considered after 6–12 months on treatment.

Monitoring for AEs, VA and anatomy on OCT scans are recommended at each treatment visit, although, in the experts’ view, full disease activity assessments are not essential at all visits and intermediary assessment visits are not required.

After 2 years of treatment patients who have remained free of disease activity and have a dry macula at three 12-week (or longer) intervals may be considered for a switch to monitoring without treatment at the discretion of the clinician. The initial monitoring review interval needs to be every 4 weeks, increased gradually at the discretion of the treating clinician. Caution should be exercised in patients on treatment in their better seeing eye; in these patients long-term fixed dosing at 12-week intervals may be preferable due to the risk of the effects of recurrent disease.

### Patients switching to brolucizumab

Patients receiving ongoing anti-VEGF therapy who require treatment discontinuation due to treatment failure should be managed in accordance with The Royal College of Ophthalmologists (RCOphth) commissioning guidance for nAMD (section 10.5 treatment discontinuation) [34]. The same criteria are expected to apply for brolucizumab as for other licensed anti-VEGF agents. NICE guidance for brolucizumab in nAMD (TA672) states “criteria for stopping should include persistent deterioration in visual acuity and identification of anatomical changes in the retina that indicate inadequate response to therapy” [33].

There are two groups of patients in whom switching to brolucizumab may be considered: first, those who remain with active disease and deteriorating VA despite anti-VEGF therapy less than every 8 weeks and, second, those where disease is controlled but in whom it is not possible to extend treatment intervals to a period that the patient finds acceptable. For the former group, it is recommended that treatment is administered, and the same interval maintained, with the hope that there will be an improved response. However, it is also important to remember that based on the results from the MERLIN study [51], the minimum injection interval with brolucizumab after the loading phase is 8 weeks. It is uncertain whether three initiation doses should be administered or treat-and-extend started immediately. Providing patients are monitored after each injection and exhibit no signs of IOI, the experts view is that loading can be used with caution. In patients who do exhibit signs of IOI, further loading should be reconsidered at the discretion of the treating clinician with further risks vs. benefits discussed with the patient. For those who have had a good response but cannot be extended satisfactorily then it is recommended that treatment is maintained at the same interval to confirm stability. Once stability is achieved, the usual treat-and-extend pattern may be continued. If there is no significant difference in the frequency of required injections, and the disease activity demands an interval of less than 8 weeks, switching back to the previous agent maybe appropriate on account of the small increased risk of IOI and current prescribing advice.

## Conclusion

Brolucizumab received marketing authorisation for the treatment of patients with nAMD based on findings from the phase 3 trials, global, head-to-head HAWK and HARRIER clinical trials. Brolucizumab offers clinicians an important anti-VEGF therapy in the treatment armamentarium that could be of benefit to overcoming and addressing the unmet needs and challenges currently faced by patients and medical retina services. This article provides HCPs with a collation of insights, guidance and resources to help support all ophthalmologic healthcare professionals (HCPs) with the necessary tools to deliver brolucizumab in their local service in the face of current and projected growth in demand for retina care.
